# Atypical Ubiquitylation in Yeast Targets Lysine-less Asi2 for Proteasomal Degradation*

**DOI:** 10.1074/jbc.M114.600593

**Published:** 2014-12-09

**Authors:** Mirta Boban, Per O. Ljungdahl, Roland Foisner

**Affiliations:** From the ‡ Max F. Perutz Laboratories, Department of Medical Biochemistry, Medical University Vienna, A-1030 Vienna, Austria and; the § Department of Molecular Biosciences, Wenner-Gren Institute, Stockholm University SE-S-10691 Stockholm, Sweden

**Keywords:** Endoplasmic Reticulum-associated Protein Degradation (ERAD), Nuclear Envelope, Proteasome, Protein Degradation, Saccharomyces cerevisiae, Ubiquitin, E2 Ubiquitin-conjugating Enzymes (Ubc6, Ubc7), E3 Ubiquitin Ligase (Doa10), Inner Nuclear Membrane, Polytopic Membrane Proteins

## Abstract

Proteins are typically targeted for proteasomal degradation by the attachment of a polyubiquitin chain to ϵ-amino groups of lysine residues. Non-lysine ubiquitylation of proteasomal substrates has been considered an atypical and rare event limited to complex eukaryotes. Here we report that a fully functional lysine-less mutant of an inner nuclear membrane protein in yeast, Asi2, is polyubiquitylated and targeted for proteasomal degradation. Efficient degradation of lysine-free Asi2 requires E3-ligase Doa10 and E2 enzymes Ubc6 and Ubc7, components of the endoplasmic reticulum-associated degradation pathway. Together, our data suggest that non-lysine ubiquitylation may be more prevalent than currently considered.

## Teaser

**Background:** Atypical degradative polyubiquitylation on non-lysine residues has only been reported in metazoans.

**Results:** Lysine-less mutant of Asi2 inner nuclear membrane protein is ubiquitylated and targeted to proteasomes in a Doa10-, Ubc6-, and Ubc7-dependent manner.

**Conclusion:** Well characterized enzymes of the endoplasmic reticulum-associated degradation pathway can catalyze atypical ubiquitylation.

**Significance:** Atypical degradative ubiquitylation is not restricted to metazoans and represents an unexplored process in yeast.

## Introduction

Protein degradation by the proteasomes is a key step in processes such as protein quality control and cell cycle progression (1, 2). Proteins are targeted to the proteasome by polyubiquitylation, a post-translational modification with a conserved 76-amino acid protein ubiquitin (3). Ubiquitylation is mediated by three classes of enzymes, ubiquitin-activating enzyme (E1), ubiquitin-conjugating enzyme (E2), and ubiquitin protein ligase (E3). The C-terminal Gly^76^ carboxyl group of ubiquitin is first activated in an ATP-dependent reaction and bound to the active cysteine of E1 by a thioester bond. The activated ubiquitin is then transferred to an E2, forming a thioester bond between the ubiquitin C terminus and the catalytic cysteine of the E2. Finally, and almost exclusively, ubiquitin is transferred to substrate proteins in an E3-dependent reaction, forming an amide (isopeptide) bond between the C terminus of ubiquitin and the ϵ-amino group of a substrate lysine. To build polyubiquitin chains that serve as effective targeting signals for proteasomal degradation, members of the C-terminal group of additional ubiquitin molecules are covalently linked to an ubiquitin molecule already attached to the substrate. This occurs via the formation of an isopeptide bond to an internal lysine residue in ubiquitin, most commonly to Lys^48^ (3).

As just described, canonical protein ubiquitylation primarily occurs on lysine residues. Ubiquitylation at alternative acceptor sites has been observed in rare cases, including the attachment of ubiquitin to a substrate cysteine sulfhydryl group via thioester bond (4–6), to serine and threonine hydroxyl groups via ester bond (7–10), and to the N termini of proteins (11). However, it is unclear how frequently such noncanonical ubiquitylation is employed as a targeting signal for protein degradation. Moreover, non-lysine ubiquitylation of protein degradation substrates has only been reported in cells from metazoan organisms and viral systems (4, 7–11), and it remains unclear whether this atypical modification is an evolutionary remnant of development that is absent in more highly selected single cell eukaryotes, such as yeast.

Asi2 is an inner nuclear membrane protein in yeast *Saccharomyces cerevisiae* that functions as a negative regulator of amino acid induced Ssy1-Ptr3-Ssy5 sensor signaling pathway (12, 13). In the absence of amino acids, Asi2 together with two other inner nuclear membrane proteins, Asi1 and Asi3, prevents promoter binding of transcription factors Stp1 and Stp2 (14, 15). We recently found that Asi2 is turned over via ubiquitin-proteasomal pathway in the nucleus involving E3 ubiquitin ligase Doa10 and E2 ubiquitin-conjugating enzymes Ubc6 and Ubc7 (16).

The ubiquitylation components Doa10-Ubc6-Ubc7 are best known for their role in the well characterized ER-associated degradation (ERAD)^2^ pathway that targets misfolded, as well as some normally folded proteins for degradation in the proteasome (17). E3 ligase Doa10 is an integral membrane protein of the ER and inner nuclear membrane (18, 19) that functions with E2 enzymes Ubc6 and Ubc7 (18). Ubc6 is also an integral membrane protein, whereas Ubc7 is tethered to the membrane via interaction with a transmembrane protein Cue1 (20). Several E2 enzymes undergo autoubiquitylation (21–24). In the case of Ubc7, a polyubiquitin chain can assemble on its catalytic cysteine residue and serve as a proteasomal degradation signal under circumstances when Ubc7 levels exceed that of its binding partner Cue1 (25).

Components of the ERAD pathway are conserved from yeast to mammals; however, in mammals, the machinery is more complex and includes additional components (26). In mammals, several substrates of the ERAD E3 ligase HRD1 were found ubiquitylated on Ser/Thr residues (8, 9). In yeast, ubiquitylation of protein degradation substrates on unconventional residues has not been reported. It is not known whether ubiquitylation machinery of yeast ERAD pathway is able to ubiquitylate substrates on non-lysine residues or whether this activity is a feature of more complex organisms.

In this study, we report that a fully functional lysine-less mutant of yeast protein Asi2 is ubiquitylated on unconventional acceptor sites and targeted for proteasomal degradation in a Doa10-Ubc6-Ubc7-dependent manner. Our study provides the first report for non-lysine ubiquitylation of a protein degradation substrate in a single cell eukaryote and indicates that components of yeast ERAD pathway can ubiquitylate substrates on unconventional acceptor sites. Together the data suggest that protein ubiquitylation on non-lysine residues may be more common than currently recognized. The finding that non-lysine ubiquitylation in yeast can target proteins for proteasomal degradation opens up enhanced opportunities to examine the biological significance of noncanonical ubiquitylation.

## EXPERIMENTAL PROCEDURES

### 

#### 

##### Yeast Growth Media

Standard yeast culture media such as yeast extract-peptone-dextrose (YPD) medium, ammonia-based synthetic minimal dextrose (SD) medium, and ammonia-based synthetic complex dextrose (SC) medium were prepared as described (27). Sensitivity to l-azetidine-2 carboxylic acid (AzC) was examined on SD medium containing 1 mm AzC, 1.3 mm l-leucine, and 1 mm l-glutamic acid. Cells were grown at 30 °C unless indicated otherwise.

##### Yeast Strains

Yeast *S. cerevisiae* strains used are listed in Table 1. All strains except *cim3-1* strains (CAY220 and PLY1348) are isogenic descendants of the S288c-derived strain AA255/PLY115 (28).

**TABLE 1:** **Yeast strains used in the study**

Yeast strain	Genotype	Reference
CAY220	*MAT*α *ura3-52 leu2*Δ*1ssd1cim3-1*::*RPT6*+	Ref. 44
MBY159	*MAT*α *ura3-52 leu2-3,112 lys2*Δ*201 asi2*Δ::*kanMX*	Ref. 16
MBY160	*MAT*α *ura3-52 leu2-3,112 lys2*Δ*201 asi2*Δ::*kanMX ubc6*Δ::*LEU2*	Ref. 16
MBY161	*MAT*α *ura3-52 leu2-3,112 lys2*Δ*201 asi2*Δ::*kanMX ubc7*Δ::*LEU2*	Ref. 16
MBY163	*MAT*α *ura3-52 lys2*Δ*201 asi2*Δ::*kanMX*	Ref. 16
MBY165	*MAT*α *ura3-52 lys2*Δ*201 asi2*Δ::*kanMX doa10*Δ::*natMX*	Ref. 16
PLY1348 (CMY763)	*MAT*α *ura3-52 leu2*Δ*1 ssd1 cim3-1*	Ref. 29
PLY1632	*MATa ura3-52 ssy5*Δ::*natMX asi2*Δ::*hisG*	Per Ljungdahl laboratory

##### Plasmids

All plasmids are listed in Table 2, and sequences of primers used for construction are listed in Table 3. Plasmid pMB117 was constructed in several steps. First, a DNA fragment (A) was constructed by PCR using primers prMB544–554 and prMB562. DNA fragments B and C were amplified by PCR from pMS1 using primers prMB538 and prMB555 (B) and prMB539 and prMB540 (C). Plasmid pMB117 was created using homologous recombination by co-transforming a Ura^−^ yeast strain with XbaI/BseRI-cut pMS1 and DNA fragments A, B, and C, followed by selection for Ura+ colonies. pMB122 was created by ligating a large fragment from BsrGI-cut pMS1 with the small fragment of similarly cut pPL741 (*ASI2*/pCT3 library, Ljungdahl laboratory). pMB123 was created by ligating a small fragment of XhoI- and NotI-cut pMB117 and a large fragment of similarly cut pMB3.

**TABLE 2:** **Plasmids used in the study**

Plasmid	Description	Reference
pMB03	Asi2-3HA/2 μ*URA3*	Ref. 15
pMB117	Asi2_K-less_-3HA/ CEN *URA3*	This study
pMB122	Asi2/CEN *URA3*	This study
pMB123	Asi2_K-less_-3HA/2μ *URA3*	This study
pMB128	Untagged Asi2/2μ *URA3*	This study
pMS1	Asi2-3HA/CEN *URA3*	Ref. 16
pRS316	CEN *URA3*	Ref. 45
Yep181-*CUP1*-myc-Ub/ *LEU2* 2μ	Ubiquitin overexpression plasmid	Helle Ulrich laboratory

**TABLE 3:** **Primers used in this study to construct plasmids**

Primer	Sequence
prMB538	AGAACCAAATATCCTGGCAAATCTCCAGTATCCCACGATTTCGATTATAAAATCATTCTCTGAAAAGGTCA
prMB539	GGGTTTTTCTTCGTATTGTGTCTATTATTCACTGTTATTAGAAGATATAGAGGTGTCCATCGAATGTTGGT
prMB540	TTATGCTTCCGGCTCGTATG
prMB544	ATAATCGAAATCGTGGGATACTGGAGATTTGCCAGGATATTTGGTTCTGG
prMB545	GTATCCCTATATGCTATTAATGTCGTGCCACCAGAACCAAATATCCTGGC
prMB546	TTAATAGCATATAGGGATACTGGTAGATTAGGGTTGCTTGGAAGGTTTCA
prMB547	ACAGGTGAAGAGTAAAATACAATGATGTTATGAAACCTTCCAAGCAACCC
prMB548	GTATTTTACTCTTCACCTGTGATAAGACATATTATGAGAAGTAGAGATGG
prMB549	AGTCTTATCCAATTTAAGTTGGGTTCATTGCCATCTCTACTTCTCATAAT
prMB550	AACTTAAATTGGATAAGACTAATGTTCGCTAGAGCTTTCGAGCTATTTGT
prMB551	GCTAAATAGATCAGAATGGTAGAGACTCTGACAAATAGCTCGAAAGCTCT
prMB552	ACCATTCTGATCTATTTAGCTTATGGCGTAAGTGGTACCGTTTACATGGT
prMB553	AGACACAATACGAAGAAAAACCCAGCGGTAACCATGTAAACGGTACCACT
prMB554	TTTTTCTTCGTATTGTGTCTATTATTCACTGTTATTAGAAGATATAGAGG
prMB555	GAACATTCTGAGGCCGGACC
prMB562	TCGATGGACACCTCTATATCTTCTAATAAC
KR87asi2fw	GCTTCTAGACATCGCCGCA
KR66asi2rev	GTGAGAACCTTTTCCAATATGATGCA
TAF10fwd	ATATTCCAGGATCAGGTCTTCCGTAGC
TAF10rev	GTAGTCTTCTCATTCTGTTGATGTTGTTGT

##### RNA Isolation and Real Time PCR

mRNA levels were analyzed as described in Ref. 16 using primer pairs: KR88asi2fwd and KR66asi2rev and TAF10fwd and TAF10rev (Table 3). Assays were conducted in triplicates on a Corbett Research RotorGene machine.

##### Cycloheximide Chase and Immunoblot Analyses

Cycloheximide chase and immunoblot analyses were performed as described in Ref. 16. Immunoblotting was performed by antibodies: anti-HA (12CA5, 1/1000, a gift from Ogris laboratory, Max F. Perutz Laboratories, Vienna, Austria), anti-Pgk1 (22C5, Invitrogen, 1/20,000), and anti-Dpm1 (5C5, Molecular Probes, 1/500), IRDye®-conjugated secondary antibody (LI-COR). Signal intensity of immunoreactive bands was quantified by Odyssey® infrared imaging system (LI-COR Biosciences). The sum of the signal intensities of both Asi2-immunoreactive bands was normalized to the signal of stable protein control Dpm1 or Pgk1.

##### Protein Ubiquitylation Assay

Ubiquitylation assay was performed as described in Ref. 16 with some modifications. Overnight cultures (28 °C) of yeast cells were diluted to *A*_600_ 0.27–0.30 in selective SC medium containing 100 μm CuSO_4_ and incubated for 2 h at 28 °C, followed by 2 h at 37 °C. Approximately 25 *A*_600_ of cells was harvested and resuspended in 450 μl of ice-cold water. 150 μl of ice-cold 50% TCA was added and incubated 15 min on ice. The cells were broken using glass beads, and samples were incubated for 10 min on ice. Protein was precipitated by 10 min of centrifugation (13,000 rpm, 4 °C), and pellet was washed with 750 μl of cold Tris. Samples can be frozen at −80 °C until further processing that is described in Ref. 16. Immunoprecipitation was performed using anti-HA affinity matrix (clone 3F10, Roche 11 815 016 001) and immunoblotting using anti-HA 12CA5 antibody (1/1000, a gift from Ogris lab, Max F. Perutz Laboratories), anti-ubiquitin P4D1 antibody (1/1000, Santa Cruz), and goat anti-mouse HRP (Jackson Immunoresearch), H+L specific (115-035-003) or light chain-specific (115-035-174) secondary antibody. Immunoreactive bands were visualized using enhanced chemiluminescence detection and x-ray films.

## RESULTS

### 

#### 

##### Lysine-less Asi2 Is Degraded via Ubiquitin-Proteasome Pathway

**FIGURE 1.**
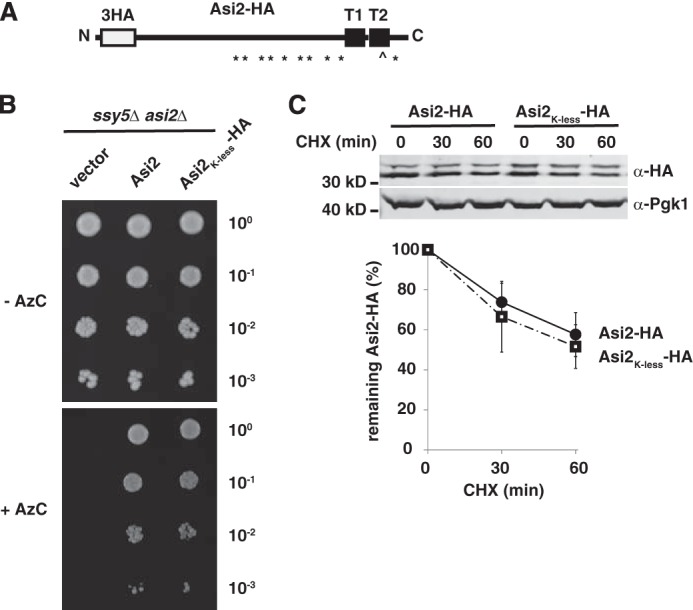
**Characterization of lysine-less Asi2 mutant.** *A*, schematic representation of HA epitope-tagged Asi2 (Asi2-HA). Epitope tag (*3HA*), transmembrane segments (*T1* and *T2*), and the positions cysteine ( ) and lysine (*) residues are indicated. *B*, Asi2_K-less_-HA is functional. Functionality of Asi2_K-less_-HA was examined in a growth-based assay. 10-fold dilutions of *ssy5*Δ *asi2*Δ (PLY1632) strain carrying plasmids pRS316 (vector), pMB122 (Asi2), or pMB117 (Asi2_K-less_-HA) were spotted on solid SD medium supplemented with the toxic proline analog AzC. AzC is transported into cells by Ssy1-Ptr3-Ssy5 sensor-regulated amino acids permeases (43). Asi2 exerts a negative regulatory function required to prevent ectopic expression of amino acid permeases in cells lacking a functional Ssy1-Ptr3-Ssy5 sensor (*ssy5*Δ) (15). Consequently, the *ssy5*Δ*asi2*Δ mutant is sensitive to AzC. Expression of Asi2 or Asi2_K-less_-HA equally restored repression of amino acid permease gene expression, hence conferring AzC resistance, indicating that the Lys-less Asi2 retains full biological activity (15). *C*, Asi2_K-less_-HA is similarly unstable to Asi2-HA. Levels of Asi2_K-less_-HA (pMB117) and Asi2-HA (pMS1) expressed in an *asi2*Δ strain (MBY163) were examined by CHX chase. Protein levels in extracts harvested at the indicated time points after CHX addition were assayed by immunoblotting with anti-HA and anti-Pgk1 antibodies. Pgk1 is a stable protein used as a control. The graph represents percentages of remaining protein after CHX addition. Average values and standard deviations of three independent sets of samples are shown. Protein half-life was 76 min (Asi2-HA) and 61 min (Asi2_K-less_-HA).

We have recently shown that inner nuclear membrane protein Asi2 is turned over in the nucleus by the ubiquitin-proteasome system involving E3 ligase Doa10 and E2 enzymes Ubc6 and Ubc7 (16). Asi2 is a 33-kDa protein consisting of two membrane-spanning segments and a 26-kDa N-terminal domain oriented toward the nucleoplasm (15) (Fig. 1*A*). To further examine the mechanism of Asi2 degradation and to identify target sites for ubiquitylation, we constructed an HA epitope-tagged Asi2 mutant in which all 10 lysine residues (Fig. 1*A*) were substituted by arginine (Asi2_K-less_-HA), using site-directed mutagenesis. Asi2_K-less_-HA mutant is functional, as shown by a growth-based assay (for details see Fig. 1*B*). We analyzed the stability of Asi2_K-less_-HA and Asi2_WT_-HA in the presence of the translation inhibitor cycloheximide. Cycloheximide (CHX) was added to growing yeast cultures, and Asi2 levels were analyzed by immunoblotting at the indicated time points. On polyacrylamide gels Asi2 resolves as two bands, suggesting a post-translational modification; however, the nature of the modification is unknown (15). Unexpectedly, the lysine-less mutant Asi2_K-less_-HA was degraded at a similar rate as Asi2_WT_-HA, indicating that both proteins are similarly turned over (Fig. 1*C*).

**FIGURE 2.**
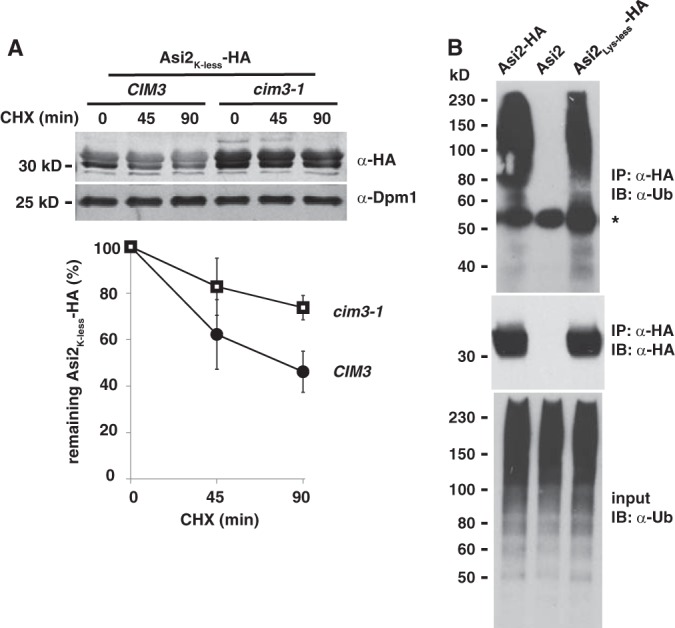
**Asi2_K-less_-HA is degraded by the ubiquitin-proteasome system.** *A*, Asi2_K-less_-HA is stabilized in proteasomal mutant *cim3-1*. Protein stability of Asi2_K-less_-HA (pMB117) expressed in a *CIM3* (CAY220) and *cim3-1* thermosensitive mutant (PLY1348) was examined by CHX chase. Immunoblotting was performed with anti-HA and anti-Dpm1 antibodies. Dpm1 is a stable protein control. Data are presented as described in Fig. 1. Asi2_K-less_-HA half-life was 74 min (*CIM3*) and 227 min (*cim3-1*). *B*, Asi2_K-less_-HA is polyubiquitylated. Immunoprecipitation (*IP*) of HA-tagged Asi2 (pMB3), Asi2_K-less_-HA (pMB123), or untagged Asi2 (pMB128) expressed in *cim3-1* strain (PLY1348) carrying ubiquitin overexpression plasmid (myc-Ub/LEU2 2μ) was performed using anti-HA antibody (*IP:* α-*HA*). A *cim3-1* mutant strain was used to enrich ubiquitylated species. Lysates from cells expressing untagged Asi2 (pMB128) were used as a control for nonspecific immunoprecipitation. Immunoblot (*IB*) analysis was done using anti-HA (α-*HA*) and anti-ubiquitin (α-*Ub*) antibodies. An *asterisk* marks a nonspecific band presumably arising because of cross-reactivity of secondary anti-mouse antibody with the rat anti-HA heavy chain used in immunoprecipitation.

To test whether Asi2_K-less_-HA is a substrate for proteasomal degradation as shown for wild-type Asi2 (16), we examined Asi2_K-less_-HA stability in a *cim3-1* strain that carries a thermosensitive allele of *RPT6* (*CIM3*) encoding an ATPase of the proteasomal lid (29, 30). Asi2_K-less_-HA was stabilized in the *cim3-1* mutant grown at a restrictive temperature (Fig. 2*A*), indicating that Asi2_K-less_-HA is indeed targeted to the proteasome for degradation.

Because most proteins are targeted to the proteasome following polyubiquitylation, we examined whether Asi2_K-less_-HA is polyubiquitylated. To enrich ubiquitylated protein species, we used a *cim3-1* mutant with impaired proteasomal function and ubiquitin overexpression from a plasmid. Anti-ubiquitin immunoblot analysis of the immune-precipitated Asi2_K-less_-HA revealed the presence of high molecular weight bands (Fig. 2*B*), indicating that Asi2_K-less_-HA is modified by polyubiquitylation. Because all lysine residues in Asi2_K-less_-HA have been mutated to arginine, Asi2_K-less_-HA is ubiquitylated on unconventional residues.

##### Lysine-free Asi2 Is Ubiquitylated on Ser/Thr Residues

**FIGURE 3.**
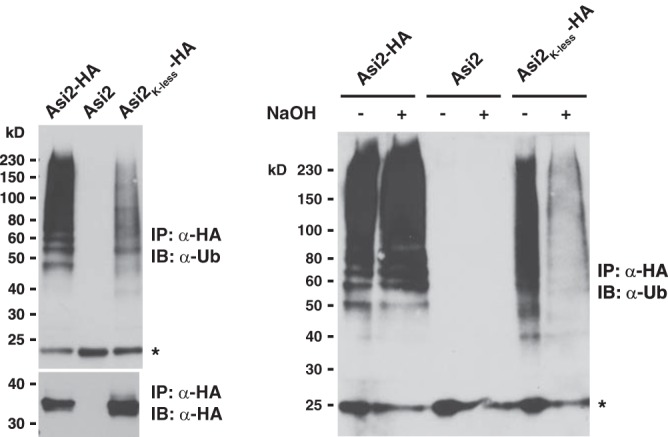
**Polyubiquitin modification of Asi2_K-less_-HA is sensitive to high pH.** Extracts from *cim3-1* strain (PLY1348) carrying the ubiquitin overexpression plasmid (myc-Ub/LEU2 2μ) and Asi2-HA (pMB3), untagged Asi2 (pMB128, control for unspecific immunoprecipitation), or Asi2_K-less_-HA (pMB123) were prepared. HA-tagged proteins were immunoprecipitated using anti-HA antibodies (*IP:* α-*HA*, *left panel*). Ten microliters of eluate was incubated with 10 μl of 300 mm NaOH or 10 μl of water (control) for 2 h at 37 °C and analyzed by immunoblotting (*right panel*) with anti-ubiquitin antibodies (*IB*, α-*Ub*). An *asterisk* marks a nonspecific band.

Next, we sought to determine which residues are ubiquitylated in lysine-free Asi2 mutants. Ubiquitin can be linked to protein Ser/Thr residues by an ester bond (7). Asi2 possesses 22 serine and 20 threonine residues. Unlike the typical isopeptide bond between a protein lysine residue and ubiquitin C-terminal carboxyl group, the ester bond between the protein serine/threonine residue and ubiquitin is sensitive to high pH (7, 8). Thus, we examined the sensitivity of Asi2 polyubiquitylation to high pH. Immunoprecipitated Asi2-HA and Asi2_K-less_-HA were incubated with 150 mm NaOH and analyzed by immunoblotting (Fig. 3). The alkaline treatment removed the polyubiquitin signal of Asi2_K-less_-HA (Fig. 3, *right panel*, *lanes 5* and *6*) but not that of Asi2_WT_-HA (Fig. 3, *right panel*, *lanes 1* and *2*). Thus, unlike wild-type Asi2, Asi2_K-less_ lacking all lysine residues is mainly ubiquitylated at Ser/Thr residues by ester bonds.

Additionally however, ubiquitin may be linked to cysteine residues via a thioester bond. Asi2 possesses only one cysteine residue at the position 262. Thioester bonds are disrupted in reducing buffers containing β-mercaptoethanol; however, the polyubiquitin signal of Asi2_K-less_-HA did not vanish in protein samples containing 2% β-mercaptoethanol (Fig. 2*B*). These data indicate that the cysteine is not ubiquitylated at all or not the only ubiquitylated residue in Asi2_K-less_-HA. Moreover, sequence analysis predicts that Cys^262^ is located within a hydrophobic region spanning the membrane (Fig. 1*A*), making it an unlikely site of ubiquitylation. Thus, although we cannot completely exclude that Asi2_K-less_ is also ubiquitylated on the Cys^262^ residue and/or on the N terminus, our data indicate that Asi2_K-less_ is ubiquitylated on Ser/Thr residues.

##### Efficient Degradation of Lysine-less Asi2 Requires E3 Ligase Doa10 and E2 Enzymes Ubc6 and Ubc7

**FIGURE 4.**
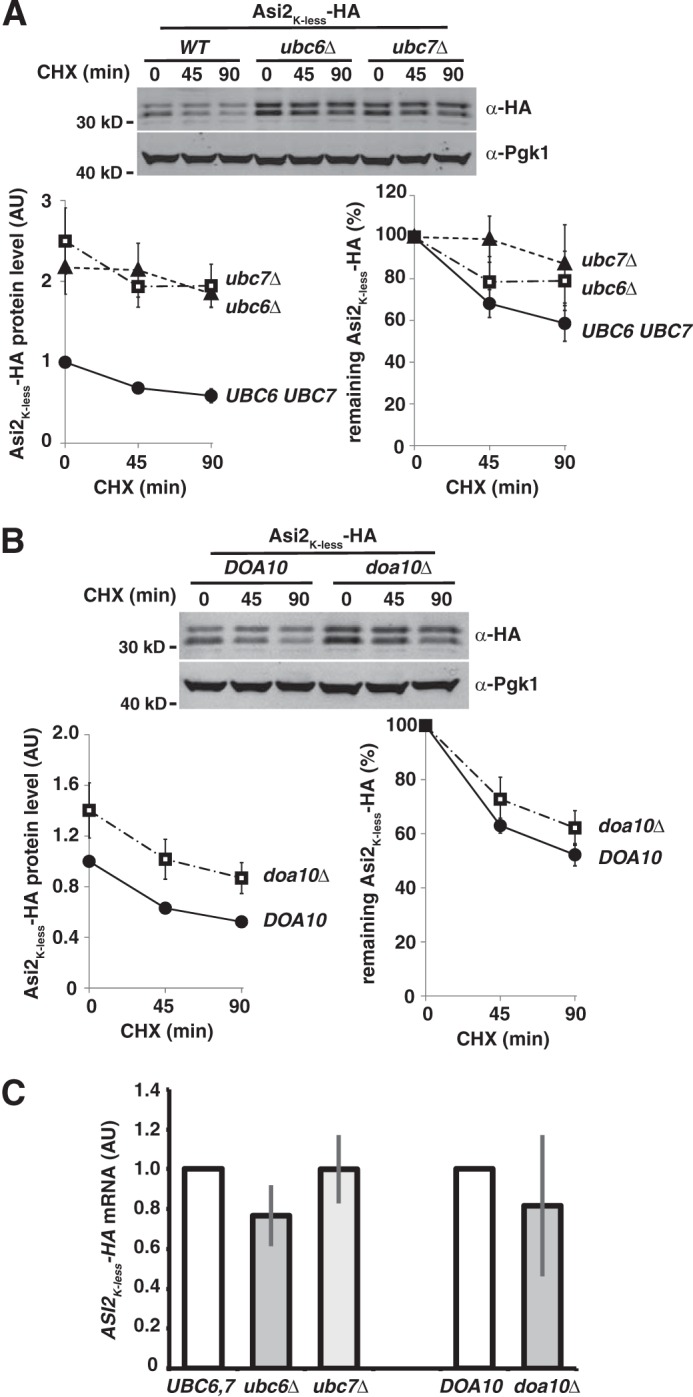
**Asi2_K-less_-HA exhibits enhanced stability in cells lacking Ubc6, Ubc7 or Doa10.** *A*, the stability of Asi2_K-less_-HA (pMB117) expressed in WT (MBY159), *ubc6*Δ (MBY160), and *ubc7*Δ (MBY161) strains. Asi2_K-less_-HA half-life was 109 min (WT), 217 min (*ubc6*Δ), and 357 min (*ubc7*Δ). *B*, the stability of Asi2_K-less_-HA (pMB117) expressed in WT (MBY163) and *doa10*Δ (MBY165) strains. Asi2_K-less_-HA half-life was 92 min (WT) and 133 min (*doa10*Δ). *A* and *B*, levels of Asi2_K-less_-HA were assessed by CHX chase followed by immunoblotting with anti-HA and anti-Pgk1 antibodies, performed as described in Fig. 1. *C*, *ASI2_K-less_-HA* mRNA levels were unaffected in *ubc6*Δ, *ubc7*Δ, and *doa10*Δ mutants. Relative *ASI2_K-less_-HA* mRNA levels were determined using RT-PCR and compared in *UBC6 UBC7* strain (MBY159) *versus ubc6*Δ (MBY160) and *ubc7*Δ (MBY161) mutants and in *DOA10* (MBY163) strain *versus doa10*Δ (MBY165) mutant expressing Asi2_K-less_-HA (pMB117). The data represent relative *ASI2_K-less_-HA* mRNA concentration (arbitrary units, *AU*). Average values and standard deviations of three independent samples are shown.

The proteasomal degradation of Asi2 in the nucleus requires E2 enzymes Ubc6 and Ubc7 and E3 ligase Doa10 (16), ubiquitylation components that also function in the ERAD pathway. (16). Although several ERAD substrates of the Doa10-Ubc6-Ubc7 pathway have been identified in yeast (18, 31–34), ubiquitylation on residues other than lysine has not been reported. To test whether the Doa10-Ubc6-Ubc7 pathway is able to target non-lysine residues in Asi2, we examined Asi2_K-less_-HA levels in strains carrying *doa10*Δ, *ubc6*Δ, and *ubc7*Δ null mutations (Fig. 4). Asi2_K-less_-HA levels were significantly elevated in each of these mutants compared with respective wild-type strains. To test the possibility that the elevated levels of Asi2_K-less_-HA were due to secondary and unanticipated consequences of increased *ASI*2_K-less_-*HA* transcription, we assessed mRNA levels in *doa10*Δ, *ubc6*Δ, and *ubc7*Δ mutants using real time quantitative PCR (Fig. 4*C*). *ASI2*_K-less_ mRNA levels were similar in wild-type and all mutant strains. The results indicate that the elevated levels of Asi2 in the cells lacking Doa10 and Ubc7 are due to enhanced Asi2 protein stability rather than elevated transcription. This finding suggests that E3 ligase Doa10 and associated E2 enzymes Ubc6 and Ubc7 are able to modify protein substrates on non-lysine residues.

## DISCUSSION

In this study, we report ubiquitylation and proteasomal targeting of a lysine-less mutant Asi2. To our knowledge, lysine-less Asi2 represents the first example of a proteasomal degradation substrate in yeast that is ubiquitylated on unconventional residues. Non-lysine ubiquitylation in yeast has only been reported as a post-translational modification regulating pathways unrelated to protein degradation, for example, the trafficking of a few peroxisomal transport receptors (6, 35, 36), where correct receptor trafficking requires ubiquitylation of a conserved cysteine.

Perhaps the most important aspect of our discovery is that lysine-less Asi2 is degraded by the proteasome in a manner dependent on E3 ligase Doa10 and associated E2s Ubc6 and Ubc7. These well characterized components of the cellular ubiquitin-proteasome system are best known for their function in ERAD (17). Although several ERAD substrates of the Doa10-Ubc6-Ubc7 pathway have been identified, such as the transcription repressor MATα2 (31), E2 Ubc6 (18), kinetochore protein Ndc10–2 (32), spindle pole body protein Mps2–1 (32, 33), and plasma membrane transporter Ste6* (34), ubiquitylation on residues other than lysine has not previously been reported. Consequently, our findings suggest that atypical non-lysine ubiquitylation is common and is an underappreciated aspect of ERAD. Notably, our data show that Asi2_K-less_ is still degraded in doa10Δ mutant, indicating that an additional degradation pathway is capable of targeting Asi2_K-less_ for degradation, similar to what we previously observed for Asi2_WT_ (16). This additional pathway could function in parallel with Doa10 or become activated under conditions under which Doa10 function is impaired.

Doa10 belongs to the K3 family of γ-herpesvirus ubiquitin ligases, which is characterized by a RING-CH domain and at least two membrane-spanning segments (37). Mouse and human γ-herpesvirus K3 ligases, MHV-K3 (mouse γ-herpes virus K3 ligase) and KSHV-K3 (human Kaposi sarcoma-associated γ-herpes virus K3 ligase), block immune detection of virus-infected cells by down-regulating levels of major histocompatibility complex class I (MHC-I) molecules through ubiquitylation on cysteine (4) or serine/threonine residues (7, 38). Although KSHV-K3 mediates MHC-I internalization from the cell surface and subsequent lysosomal degradation, MHV-K3 ubiquitylates newly synthesized MHC-I in the ER membrane and targets it for proteasomal degradation (37). Interestingly, ubiquitylation of MHC-I by the MHV-K3 requires the endogenous E2 enzyme Ube2j2 (39), the mammalian homolog of yeast Ubc6 (40). There are nine human K3 homologs, including the Doa10 homolog MARCH VI (Teb4) (41). Doa10 is the only RING-CH family member in yeast (18). Notably, not all RING-CH family E3 ligases are capable of ubiquitylating substrates on non-lysine residues (41). Herr *et al.* (42) found that sequences outside the RING-CH domain of MHV-K3 determine whether and which non-lysine substrate residues can be ubiquitylated.

Our data indicate that lysine-less mutant Asi2 is ubiquitylated on Ser/Thr residues. Remarkably, wild-type Asi2 appears preferentially ubiquitylated on lysine residues, suggesting that unconventional ubiquitylation of protein quality control substrates in yeast might occur when lysine residues are not available or not readily accessible to the ubiquitylation machinery. We observed that a stronger anti-ubiquitin signal was associated with Asi2-WT-HA than with Asi2-K-less-HA (Figs. 2*B* and 3*A*), which may suggest that more or longer ubiquitin chains are associated with lysine residues in Asi2_WT_ than with non-lysine residues in Asi2_K-less_. However, we suspect that ubiquitin chains ester-bound to Asi2_K-less_-HA are partially lost during the protein preparation, which includes a step with acidic conditions. In contrast, ubiquitin linkage to Asi2_WT_ lysines by isopeptide bond is insensitive to those conditions. Similarly to Doa10, which preferentially ubiquitylates Asi2_WT_ on lysine residues, KSHV-K3 preferentially ubiquitylates MHC-I molecules on lysines, whereas MHC-I ubiquitylation on cysteine or serine residues is primarily observed in the context of a lysine-less mutant (4). In contrast, the mouse γ-herpesvirus K3 ubiquitin ligase preferentially mediates MHC-I ubiquitylation on Ser/Thr residues even when lysine residues are present in the protein (39). Thus, related E3s can have different preferences for ubiquitylation of specific residues. The significance of altered residue specificity is not clear. However, ubiquitin linked to Ser/Thr may be more resistant to cleavage by deubiquitylating enzymes (39). Also, the capacity to catalyze non-lysine ubiquitylation may facilitate control over a wider range of substrates.

To conclude, our study provides the first example of a protein degradation substrate in yeast that is modified by ubiquitin on unconventional acceptor sites. We show that ubiquitylation components Doa10-Ubc6-Ubc7 target lysine-less Asi2 for proteasomal degradation. The fact that these well characterized ubiquitylation components have a major role in ERAD suggests that non-lysine ubiquitylation may function as a previously unrecognized targeting signal in this pathway. Together our findings indicate that alternative site ubiquitylation of protein degradation substrates is not restricted to complex eukaryotes and cell-derived viruses and consequently may be much more prevalent than currently recognized. Perhaps most importantly, our data showing that in the absence of lysines alternative ubiquitylation sites are selected indicate that the targeting process is characterized by plasticity rather than rigid site-specific conjugation. The significance of this finding clearly deserves further experimental attention.

## Footnotes

2 The abbreviations used are:

ERAD: endoplasmic reticulum-associated degradation
MHC-I: major histocompatibility complex class I
AzC: l-azetidine-2 carboxylic acid
CHX: cycloheximide.
